# Apatinib inhibits glycolysis by suppressing the VEGFR2/AKT1/SOX5/GLUT4 signaling pathway in ovarian cancer cells

**DOI:** 10.1007/s13402-019-00455-x

**Published:** 2019-07-20

**Authors:** Lihua Chen, Xi Cheng, Wenzhi Tu, Zihao Qi, Haoran Li, Fei Liu, Yufei Yang, Zhe Zhang, Ziliang Wang

**Affiliations:** 1 Department of Gynecological Oncology and Department of Medical Oncology, Fudan University Shanghai Cancer Center, 270 Dong’an Road, Shanghai, 200032 China; 2 Department of Oncology, Shanghai Medical College, Fudan University, 270 Dong’an Road, Shanghai, 200032 China; 3 Department of Radiation Oncology, Shanghai General Hospital, Shanghai Jiao Tong University School of Medicine, Shanghai, 201620 China; 4 Department of Obstetrics and Gynecology, Xihua Hospital Affiliated to Shanghai Jiao Tong University School of Medicine, 1665 Kongjiang Road, Shanghai, 200092 China

**Keywords:** Ovarian cancer, Apatinib, Glucose metabolism, VEGFR2, SOX5

## Abstract

**Background:**

Apatinib is a tyrosine kinase inhibitor that targets vascular endothelial growth factor receptor-2 (VEGFR2), and has shown encouraging therapeutic effects in various malignant tumors. As yet, however, the role of apatinib in ovarian cancer has remained unknown. Here, we sought to elucidate the role of apatinib in the in vitro and in vivo viability and proliferation of ovarian cancer cells, as well as in glucose metabolism in these cells.

**Methods:**

The effects of apatinib on ovarian cancer cell viability and proliferation were assessed using Cell Counting Kit-8 (CCK-8) and colony formation assays, respectively. The expression of VEGFR2/AKT1/SOX5/GLUT4 pathway proteins was assessed using Western blotting, and glucose uptake and lactate production assays were used to detect glycolysis in ovarian cancer cells. SOX5 was exogenously over-expressed and silenced in ovarian cancer cells using expression vector and shRNA-based methods, respectively. RNA expression analyses were performed using RNA-seq and gene-chip-based methods. GLUT4 promoter activity was assessed using a dual-luciferase reporter assay. The expression of p-VEGFR2 (Tyr1175), p-AKT1 (Ser473), p-GSK3β (Ser9), SOX5 and GLUT4 in xenograft tissues was assessed using immunohistochemistry (IHC).

**Results:**

We found that apatinib inhibited the in vitro and in vivo viability and proliferation in Hey and OVCA433 ovarian cancer cells in a dose-dependent and time-dependent manner. We also found that apatinib effectively suppressed glucose uptake and lactate production by blocking the expression of GLUT4 in these cells. In addition, we found that SOX5 predominantly rescued the inhibitory effect of apatinib on GLUT4 expression by activating its promoter. Finally, we found that apatinib regulated the expression of SOX5 by suppressing the VEGFR2/AKT1/GSK3β signaling pathway.

**Conclusions:**

From our results, we conclude that apatinib suppresses the in vitro and in vivo viability and proliferation of ovarian cancer cells, as well as glycolysis by inhibiting the VEGFR2/AKT1/GSK3β/SOX5/GLUT4 signaling pathway. Apatinib may serve as a promising drug for the treatment of ovarian cancer.

**Electronic supplementary material:**

The online version of this article (10.1007/s13402-019-00455-x) contains supplementary material, which is available to authorized users.

## Introduction

Ovarian cancer is the seventh most common cancer worldwide and the leading cause of death among patients with gynecological malignancies [1]. About 70% of the cases are diagnosed at advanced stages and 75–85% of the patients relapse even after satisfactory cytoreduction and integrated adjuvant chemotherapy [2]. After different episodes of recurrent disease, patients may develop chemoresistance, with a 5-year survival < 50% [3]. Thus, there is an urgent need for novel treatment options and the identification of drugs that specifically target key molecules [4].

Angiogenesis plays a vital role in tumor growth and metastasis, and anti-angiogenic drugs may provide treatment options for patients suffering from failure of multi-line chemotherapy, such as bevacizumab, ramucirumab and apatinib. Vascular endothelial growth factor receptor-2 (VEGFR2) is mainly expressed in endothelial cells and has been found to act as a pivotal meditator of angiogenesis in some solid tumors. Recent reports have indicated that the expression of VEGFR2 may be up-regulated in solid tumor cells, and may be related to an invasive behavior and a poor clinical outcome [5–9]. Apatinib is a novel receptor tyrosine kinase inhibitor that targets VEGFR2 with a high specificity. It is an orally bioavailable agent that has shown a favorable anti-tumor efficacy with manageable side effects [10–12]. Based on the results of a multi-centered phase III clinical trial, it has been concluded that apatinib may significantly improve the survival of patients with advanced gastric cancers [13]. In a phase II clinical trial on metastatic triple-negative breast cancer, apatinib has also been found to benefit patient survival [14]. As yet, however, few studies on the therapeutic effects and mechanisms of action of apatinib in ovarian cancer have been reported. Some case reports have indicated that apatinib may be an option for patients with chemotherapy-refractory advanced epithelial ovarian cancer [15, 16]. A phase II study has shown that apatinib may be a feasible treatment option for recurrent, platinum-resistant epithelial ovarian cancer [17], and another recent phase II prospective study suggests that apatinib combined with etoposide may show promising effects with manageable toxicities in platinum-refractory ovarian cancer [18].

SRY-related high-mobility-group box 5 (SOX5) is a member of the SOX family of proteins and acts as a transcription factor involved in the regulation of embryonic development and cell fate determination [19]. SOX5 has also been found to play an important role in various types of cancer. It has, for example, been reported that SOX5 may inhibit PDGFB-induced glioma development [20]. Accumulating evidence also indicates that SOX5 may act as an oncogenic factor through interaction with yes-associated protein 1 (YAP1), thereby promoting the migration and proliferation of non-small cell lung cancer cells [21]. Furthermore, SOX5 has been found to promote glucose metabolism in type 2 diabetes patients [22].

Here, we explored the effects of apatinib on ovarian cancer cells and found that apatinib inhibited their proliferation by affecting glucose metabolism. We also found that apatinib suppressed glucose metabolism in these cells via the VEGFR2/AKT1/ GSK3β/SOX5 signaling pathway. Our data provide evidence for a putative therapeutic efficacy of apatinib in ovarian cancer.

## Materials and methods

### Cell lines and cell culture

The human ovarian cancer-derived cell lines Hey, OVCA433, SKOV3, IGROV1, OVCA420, OVCA429, ES-2, OVCAR-8 and a HUVEC (Human Umbilical Vein Endothelial Cell) cell line were purchased from the American Type Culture Collection (ATCC). All cells were maintained in Dulbecco’s modified Eagle’s medium (DMEM, HyClone, Thermo Scientific, USA), supplemented with 10% fetal bovine serum (Gibco, Life technologies, USA), 100 U/ml penicillin (Biowest, Nuaillé, France) and 100 U/ml streptomycin (Biowest, Nuaillé, France), at 37 °C in a humidified atmosphere with 5% CO_2_.

### Apatinib and cell culture treatment

Apatinib was purchased from MCE (MedChemExpress). To prepare apatinib at a concentration of 10 mM, 10 mg powder was dissolved in 2.026 ml DMSO and stored at 4 °C until use. Ovarian cell cultures were treated with different concentrations (μmol/L) apatinib for 48 h.

### Cell viability assay

To evaluate cell viability, 5 × 10^3^ ovarian cancer cells were seeded per well in 96-well plates with 100 μl culture medium. The next day, the cells were treated with different concentrations of apatinib. A Cell Counting Kit-8 (CCK-8) (Dojindo Laboratories, Kumamoto, Japan) was used to monitor cell viability after 24, 48 and 72 h, respectively, and the number of viable cells was assessed by absorbance measurement after a two-hour incubation at 450 nm using a Microplate Reader (BioTek Instruments, Winooski, VT, USA). The viability rate was calculated as experimental OD value/control OD value.

### Plasmid construction and transfection

The recombinant plasmids pENTER-SOX5, pENTER-AKT1 and pENTER-VEGFA containing human full-length cDNA sequences of SOX5, AKT1 and VEGFA, respectively, were purchased from Vigene Biosciences (Jinan, China). The expression of SOX5 was silenced using two specific shRNAs obtained from Vigene Biosciences (Jinan, China). Both Hey and OVCA433 cells were transfected using a transfection reagent (Hieff TransTM Liposomal Transfection Reagent, YEASEN) according to the manufacturer’s instructions. Control cell lines were generated through transfection with an empty vector following the same protocol. SiRNAs to silence the expression of VEGFR2 and AKT1 were purchased from Guangzhou RiboBio Co.LTD (Guangzhou, China).

### Quantitative real-time PCR

Total RNAs of Hey and OVCA433 cells were isolated using Trizol reagent (Invitrogen, Life technologies, USA) and reverse transcribed into cDNA using a PrimeScript™ RT Master Mix (Perfect Real Time; Takara Biotechnology, Shiga, Japan). Real-time PCR was carried out in an Applied Biosystems Prism 7900 system (Applied Biosystems, Life technologies, USA) with TB Green™ Premix Ex Taq™ II (Tli RNaseH Plus) (Takara Biotechnology, Shiga, Japan) using the following conditions: an initial denaturation at 95 °C for 30 s, followed by 95 °C for 5 s, 55 °C for 30 s and 72 °C for 30 s, 40 cycles, after which melting curve analysis was performed to check the specificity of amplification. Each sample was tested in triplicate and Glyceraldehyde 3-phosphatedehydrogenase (GAPDH) was used in parallel reactions as internal control. Three independent experiments were carried out for final analyses using the 2-ΔΔCT relative quantification method. The primer sequences used are listed in Supplementary Table S2.

### Glycolysis assay

Cells were pretreated as indicated for 24 h, seeded in a 96-well plate and maintained overnight. Next, the culture medium was removed and the cells were washed thrice with PBS, after which glucose uptake was measured using a Glucose Uptake Colorimetric Assay kit (BioVision, USA) according to the manufacturer’s protocol. For lactate measurement, the cells were cultured in DMEM without phenol red (Hyclone, USA) for 15 h, after which the culture medium was collected. Quantification of the lactate levels was performed using a Lactate Colorimetric Assay kit (BioVision, USA) and the concentrations were normalized against the corresponding total protein level using a BCA protein assay kit (Beyotime Biotechnology).

### Western blot analysis

Western blot analyses were carried out to determine the expression levels of various proteins. Cells were pre-treated with apatinib at different concentrations or DMSO (diluent) for 48 h. Next, the cells were harvested, washed with cold 1 × PBS, and lysed in moderate RIPA lysis buffer (Beyotime Biotechnology) containing a protease inhibitor cocktail (Roche Diagnostics). Total protein concentrations were determined using a BCA protein assay kit (Beyotime Biotechnology). Equal amounts (24 μg per load) of the protein samples were subjected to SDS-PAGE and transferred to polyvinylidene fluoride (PVDF) membranes (Millipore). The resulting blots were blocked in 5% bovine serum albumin (BSA) and incubated with primary antibodies, followed by incubation with secondary antibodies conjugated with horseradish peroxidase (HRP). Protein bands were visualized using a chemiluminescent reagent (Millipore). Antibodies directed against LDHA, HK2, SOX5, VEGFA, AKT1 and β-Actin were purchased from Proteintech, and antibodies directed against VEGFR2, p-AKT1(Ser473), p-GSK3β (Glycogen synthase kinase 3 beta) (Ser9) and p-VEGFR2(Tyr1175) were purchased from Cell Signaling Technology. The antibody directed against GLUT4 was obtained from Signalway Antibody.

### Colony formation assay

Cells treated with apatinib at different concentrations or DMSO for 48 h were seeded in 6-well plates at a density of 500 cells per well. The cells were cultured with fresh medium and allowed to grow at least for 1 week before being fixed with paraformaldehyde and stained with Crystal violet (Beyotime Biotechnology), after which colonies were counted.

### RNA-seq and data analysis

Total RNA (1 μg) was isolated from Hey cells and treated with VAHTS mRNA Capture Beads (Vazyme, Nanjing, China) to enrich for polyA+ RNA before constructing RNA libraries. RNA library preparation was performed using a VAHTS mRNA-seq v2 Library Prep kit from Illumina (Vazyme, Nanjing, China). Paired-end sequencing was performed using an Illumina HiSeq 3000 apparatus at RiboBio Co., Ltd. (Guangzhou, China). For computational analysis of the RNA-seq data, sequencing reads were aligned using the spliced read aligner HISAT2, which is supplied by the Ensembl human genome assembly (Genome Reference Consortium GRCh38) as reference genome. Gene expression levels were calculated using FPKM (fragments per kilobase of transcript per million mapped reads). Gene Set Enrichment Analysis (GSEA) was used for functional gene annotation.

### Dual-luciferase reporter assay

The human GLUT4 gene promoter region was inserted into a pGL3 basic vector as pGL3-GLUT4-Promoter. 100 ng of the constructed plasmid and 7 ng Renilla luciferase control plasmid were transfected into cells in which SOX5 was overexpressed or silenced in 6-well plates. After 48 h, luciferase activities were measured using a Dual Luciferase Assay kit (Promega, Madison, WI, USA). Reporter luciferase activities were normalized to Renilla luciferase activity and then rescaled to vector control signals equal to unit 1.

### Immunohistochemistry

Immunohistochemistry (IHC) assays were carried out as described previously [23, 24]. IHC was performed on xenograft tumor sections using antibodies directed against p-VEGFR2(Tyr1175) (rabbit polyclonal antibody, Cell Signaling Technology, 1:100 dilution), p-AKT1(Ser473) (rabbit polyclonal antibody, Cell Signaling Technology, 1:100 dilution), p-GSK3β(Ser9) (rabbit polyclonal antibody, Cell Signaling Technology, 1:300 dilution), CD31 (rabbit polyclonal antibody, Abcam, 1:50 dilution), GLUT4 (rabbit polyclonal antibody, Signalway Antibody, 1:100 dilution) and SOX5 (rabbit polyclonal antibody, Proteintech, 1:100 dilution). IHC staining was scored based on both the percentage of positive tumor cells and staining intensity. Negative expression was defined as < 2+ and positive as > 2+ to < 6 + .

### In vivo tumor growth assay

All animal experiments were approved by the Ethics Committee at FUSCC. Female BALB/c nude mice (Shanghai Slac Laboratory Animal Co. Ltd., 4–6 weeks) were injected subcutaneously with Hey cells (5 × 10^6^ suspended in 0.1 ml PBS per mouse). Once reaching an average tumor volume of 100 mm^3^, mice were randomized into different groups (*n* = 5 / group), after which they were intraperitoneally injected with apatinib (50 mg/kg) or vehicle. The tumor sizes were measured using a digital caliper once every 3 days. The tumor volumes were calculated using the following equation: V = L × W^2^ × 0.52, in which V represents volume, L represents length and W represents width. One week after the last apatinib injection the mice were sacrificed and the tumors were dissected and weighed. IHC of the xenografted tumors was carried out according to the protocol described above.

### Statistical analysis

All experiments were performed in triplicate. The results were calculated using Graph Pad Prism and reported as mean ± S.E. Comparisons between controls and treated groups were determined using t test or one-way ANOVA, followed by Tukey’s multiple comparison tests. A *p* value < 0.05 was taken as statistically significant.

## Results

### Apatinib suppresses glycolysis in ovarian cancer cells

**Fig. 1:**
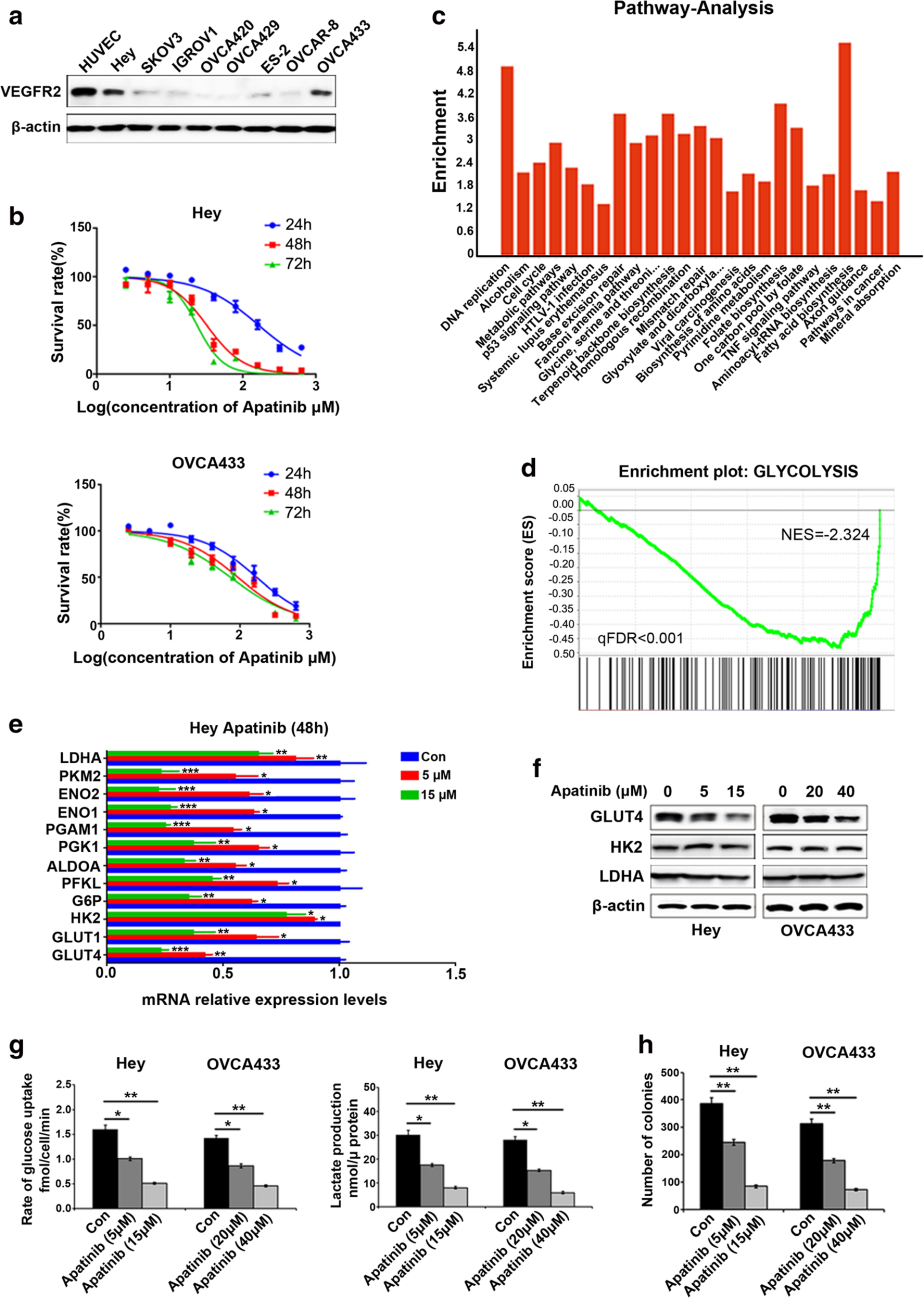
Apatinib suppresses proliferation by inhibiting glycolysis in ovarian cancer cells. **a** Expression levels of VEGFR2 in ovarian cancer cell lines and HUVEC cells. **b** Dose-dependent and time-dependent decreases in viability of Hey and OVCA433 cells compared to control cells treated with different concentrations of apatinib. **c** Gene-chip-based pathway analysis after apatinib treatment. **d** Hey cells were treated with 15 μM apatinib for 48 h, after which total RNA was extracted for RNA sequencing. Genes related to glycolysis were found to be down-regulated by apatinib (qFDR < 0.001). **e** Expression levels of common glycolysis-related genes after apatinib treatment for 48 h at 5 and 15 μM in Hey cells. **f** Western blotting showing that the expression of GLUT4 was reduced, while that of LDHA and HK2 remained unchanged compared to the corresponding controls. **g** After exposure to apatinib, both glucose uptake and lactate production were significantly reduced compared to the corresponding controls (*p* < 0.05). **h** After exposure to different concentrations of apatinib the number of ovarian cancer cell colonies decreased progressively compared to the untreated controls (*p* < 0.05)

Western blotting was used to assess VEGFR2 expression in ovarian cancer-derived and HUVEC cells. We found that, compared to SKOV3, IGROV1, OVCA420, OVCA429, OVCAR-8 and ES-2 cells, the expression of VEGFR2 in Hey and OVCA433 cells was significantly higher (Fig. 1a). Therefore, these latter two cell lines were selected for subsequent experiments. To this end, Hey and OVCA433 cells were treated with different concentrations of apatinib (0, 2.5, 5, 10, 20, 40, 80, 160, 320 or 640 μΜ) for 24, 48 and 72 h, respectively (Fig. 1b). We found that in both cell lines, apatinib treatment resulted in a decreased cell viability compared to that in controls in a dose-dependent and time-dependent manner. The half-maximal inhibitory concentration (IC50) value at 48 h for Hey and OVCA433 cells were 32.15 (95% CI 28.17–36.73) μM and 92.85 (95% CI 77.36–111.1) μM, respectively. The concentrations used in our subsequent experiments were calculated from these dose-response curves.

To assess the effect of apatinib on gene expression profiles in ovarian cancer cells we performed gene-chip assays in apatinib-treated Hey cells and control cells. Subsequent pathway analysis revealed that several of the pathways altered after apatinib treatment were related to glycolysis (Fig. 1c-d) and both qRT-PCR and Western blotting results indicated that GLUT4 was among the top factors of which the expression was altered after apatinib treatment (Fig. 1e-f**)**. To further assess the role of apatinib in glycolysis, we performed glucose uptake and lactate production assays in Hey and OVCA433 cells treated with apatinib at different concentrations (5 and 15 μM for Hey cells, and 20 and 40 μM for OVCA433 cells). We found that the glucose uptake and lactate production were significantly repressed compared to the corresponding controls in both apatinib-treated cell lines (*p* < 0.05) (Fig. 1g). Using a colony assay, we also found that the numbers of colonies formed by Hey and OVCA433 cells were decreased after treatment with different concentrations of apatinib (Fig. 1h).

### Apatinib regulates glucose metabolism by inhibiting the expression of SOX5 in ovarian cancer cells

**Fig. 2:**
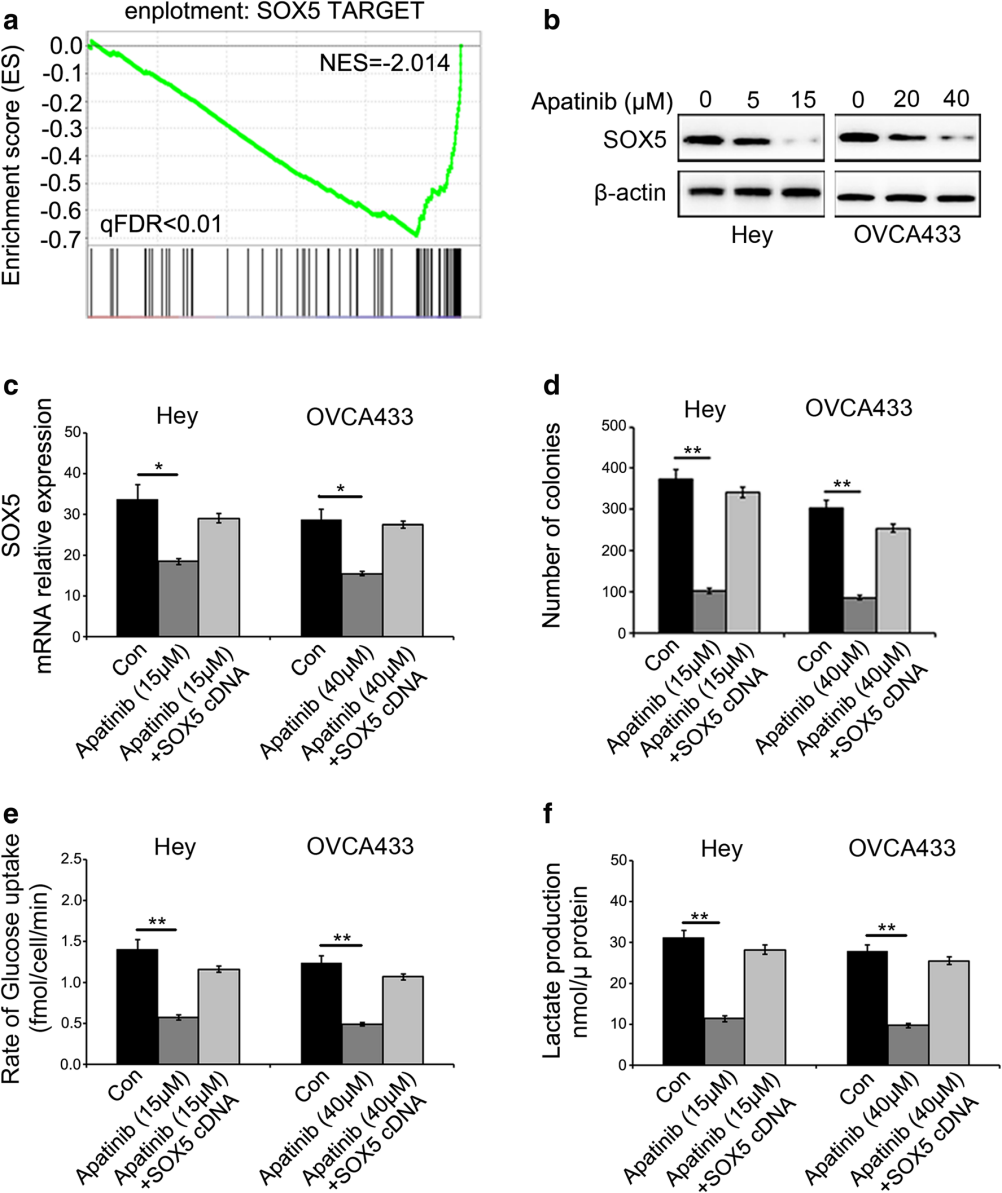
Apatinib regulates glycolysis by inhibiting SOX5 expression in ovarian cancer cells. **a** RNA sequencing results showing that SOX5 target genes are down-regulated after apatinib treatment (qFDR < 0.01). **b** Suppression of SOX5 protein levels after treatment with the given concentrations of apatinib. **c** Exogenous overexpression of SOX5 partly reversed the decline in SOX5 mRNA levels induced by apatinib. **d-f** Exogenous SOX5 overexpression rescued colony formation and glucose uptake, as also lactate production, inhibited by apatinib in ovarian cancer cells

To explore the mechanism underlying apatinib-induced glycolysis suppression, we analyzed RNA-seq data and, by doing so, identified SOX5 as a potential downstream target (Fig. 2a). Using Western blotting, we subsequently found that the expression of SOX5 in Hey and OVCA433 cells was suppressed by apatinib treatment in a dose-dependent manner (Fig. 2b). To next determine the role of SOX5 in apatinib-mediated glycolysis, we exogenously overexpressed SOX5 in apatinib-treated Hey and OVCA433 cells. We found that this exogenous SOX5 overexpression (Fig. 2c) effectively reversed the effect of apatinib on colony formation, glucose uptake and lactate production in these cells (Fig. 2d-f).

### SOX5 affects glycolysis via regulating GLUT4 promoter activity in ovarian cancer cells

**Fig. 3:**
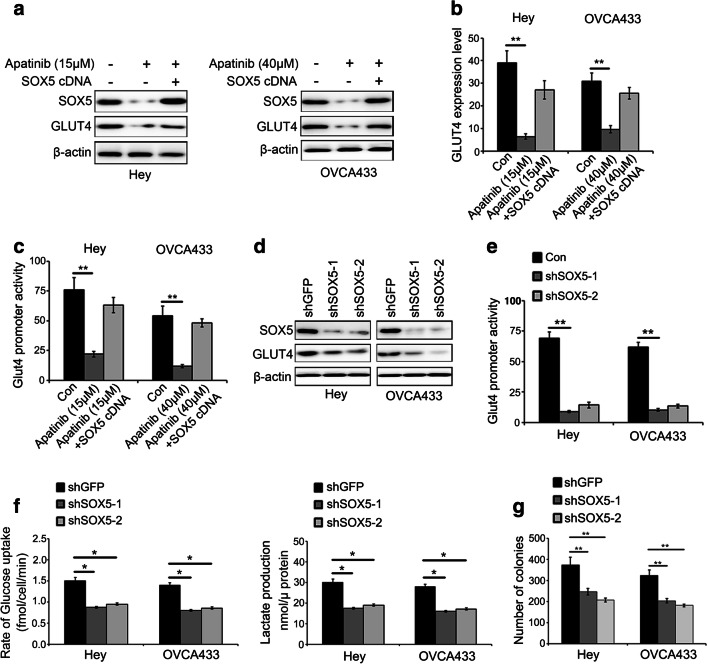
SOX5 binds to the promoter region of GLUT4 in ovarian cancer cells. **a-b** Exogenous SOX5 overexpression rescued the decline in GLUT4 expression induced by apatinib at both the mRNA and protein levels. **c** Exogenous SOX5 overexpression partly reversed the decrease in GLUT4 promoter activity caused by apatinib. **d-e** SOX5 expression knockdown inhibited the promoter activity of GLUT4. **f-g** SOX5 silencing in ovarian cancer cells reduced glucose intake, lactate production and colony formation

We also found that exogenous overexpression of SOX5 rescued the inhibitory effect of apatinib on GLUT4 expression (Fig. 3a-b, Table S1). Considering the notion that SOX5 functions as a transcription factor, we hypothesized that SOX5 may regulate the transcription of GLUT4 in response to apatinib. To test this hypothesis, we performed a dual-luciferase reporter assay and, by doing so, indeed found that SOX5 overexpression significantly rescued the reduction in GLUT4 promoter activity caused by apatinib (Fig. 3c). Additionally, we knocked down the expression of SOX5 in Hey and OVCA433 cells using shRNA and found that, concomitantly, the expression of GLUT4 was down-regulated (Fig. 3d). We also found that the GLUT4 promoter activity was decreased dramatically when SOX5 shRNA was introduced into Hey and OVCA433 cells and that SOX5 silencing led to a decreased glucose uptake, lactate production and colony formation in these cells compared to controls (Fig. 3e-g). These results indicate that apatinib suppresses glycolysis in ovarian cancer cells by inhibiting the SOX5/GLUT4 signaling pathway.

### Apatinib regulates SOX5 expression via the VEGFR2/AKT1/GSK3β signaling pathway in ovarian cancer cells

**Fig. 4:**
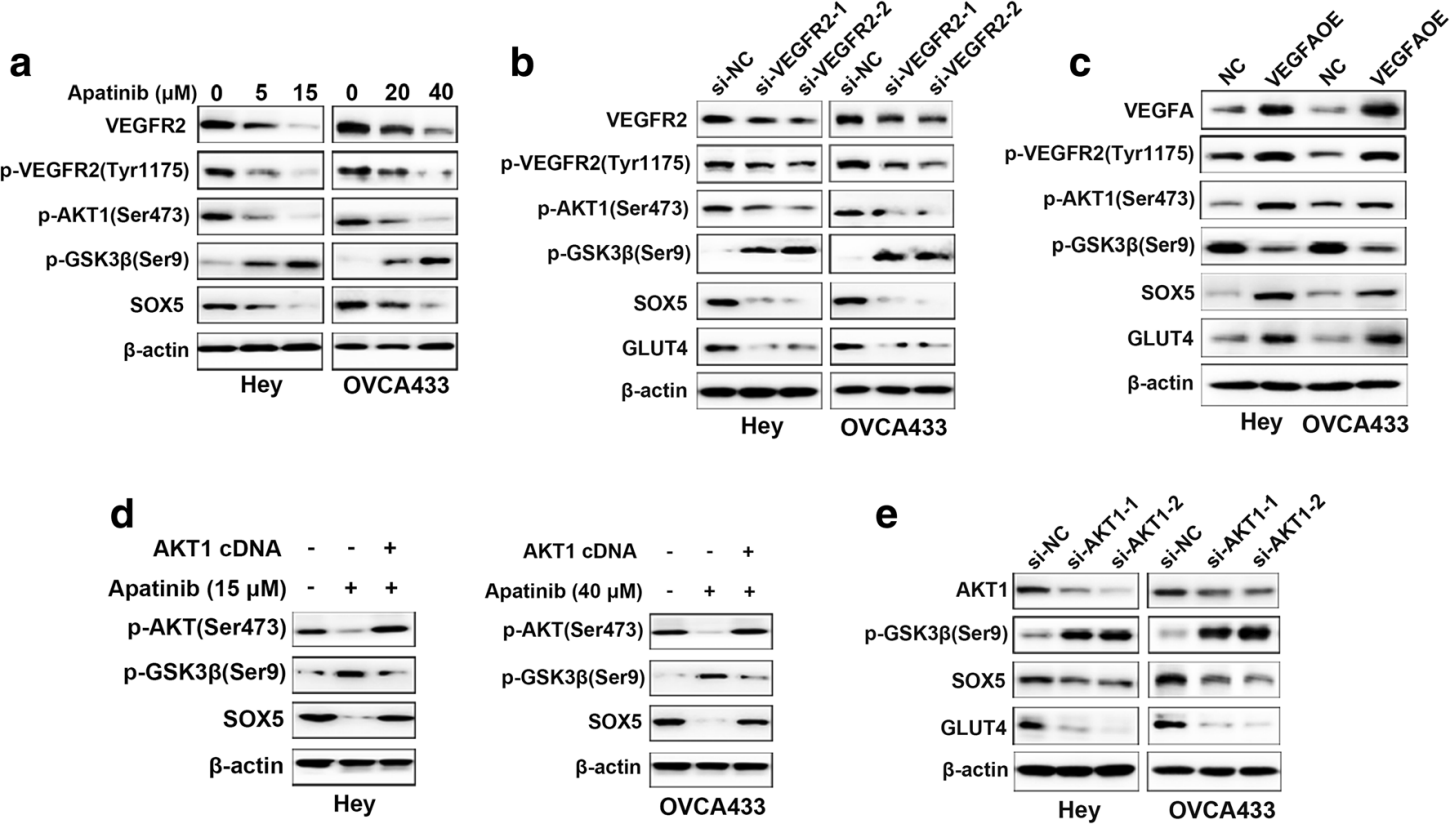
Expression levels of VEGFR2, p-VEGFR2 (Tyr 1175), p-AKT1 (Ser473), p-GSK3β (Ser9), SOX5 in cells treated with apatinib, siRNAs for VEGFR2, VEGFA cDNA or siRNAs for AKT1 or AKT1 cDNA. **a** Apatinib treatment decreased the expression of VEGFR2, p-VEGFR2(Tyr1175), p-AKT1(Ser473) and SOX5, but enhanced the expression of p-GSK3β(Ser9) compared to controls as detected by Western blotting. **b** VEGFR2 silencing resulted in similar protein changes compared to those caused by apatinib. **c** Exogenous VEGFA overexpression induced the expression of p-VEGFR2(Tyr1175), p-AKT1(Ser473), SOX5 and GLUT4, but reduced the expression of p-GSK3β (Ser9), compared to controls. The reverse was noted after VEGFR2 silencing or apatinib treatment. **d** Exogenous AKT1 overexpression rescued the effect of apatinib on SOX5 suppression and decreased the expression of p-GSK3β(Ser9). **e** AKT1 expression knockdown also resulted in an elevated expression of p-GSK3β(Ser9), and a decreased expression of SOX5 and GLUT4 in Hey and OVCA433 cells as detected by Western blotting

Using a gene-chip-based expression assay we found that apatinib may inhibit cell proliferation and metabolism by suppressing AKT1 and its downstream effectors, including MAPK and P53 (Fig. S1). Additionally, we found by Western blotting that apatinib suppressed the expression of phospho-VEGFR2(Tyr1175), phospho-AKT1(Ser473) and SOX5, but enhanced the expression of phospho-GSK3β(Ser9), compared to the corresponding controls (Fig. 4a). To further elucidate the role of VEGFR2, specific siRNAs were used to silence its expression and, by doing so, similar protein alterations compared to those observed after apatinib treatment were noted (Fig. 4b). We also found that exogenous VEGFA overexpression led to elevated expression levels of phospho-VEGFR2, phospho-AKT1, SOX5 and GLUT4, but a decreased expression level of phospho-GSK3β (Fig. 4c). Also, exogenous AKT1 overexpression was found to effectively attenuate the expression of phospho-GSK3β, but to enhance the expression of SOX5 (Fig. 4d), which is consistent with the alterations observed after AKT1 silencing (Fig. 4e). Together, the above results suggest that apatinib may inhibit SOX5 expression via the VEGFR2/AKT1/GSK3β signaling pathway to regulate glycolysis in ovarian cancer cells.

### Apatinib inhibits cell proliferation and glycolysis of ovarian cancer cells in vivo

**Fig. 5:**
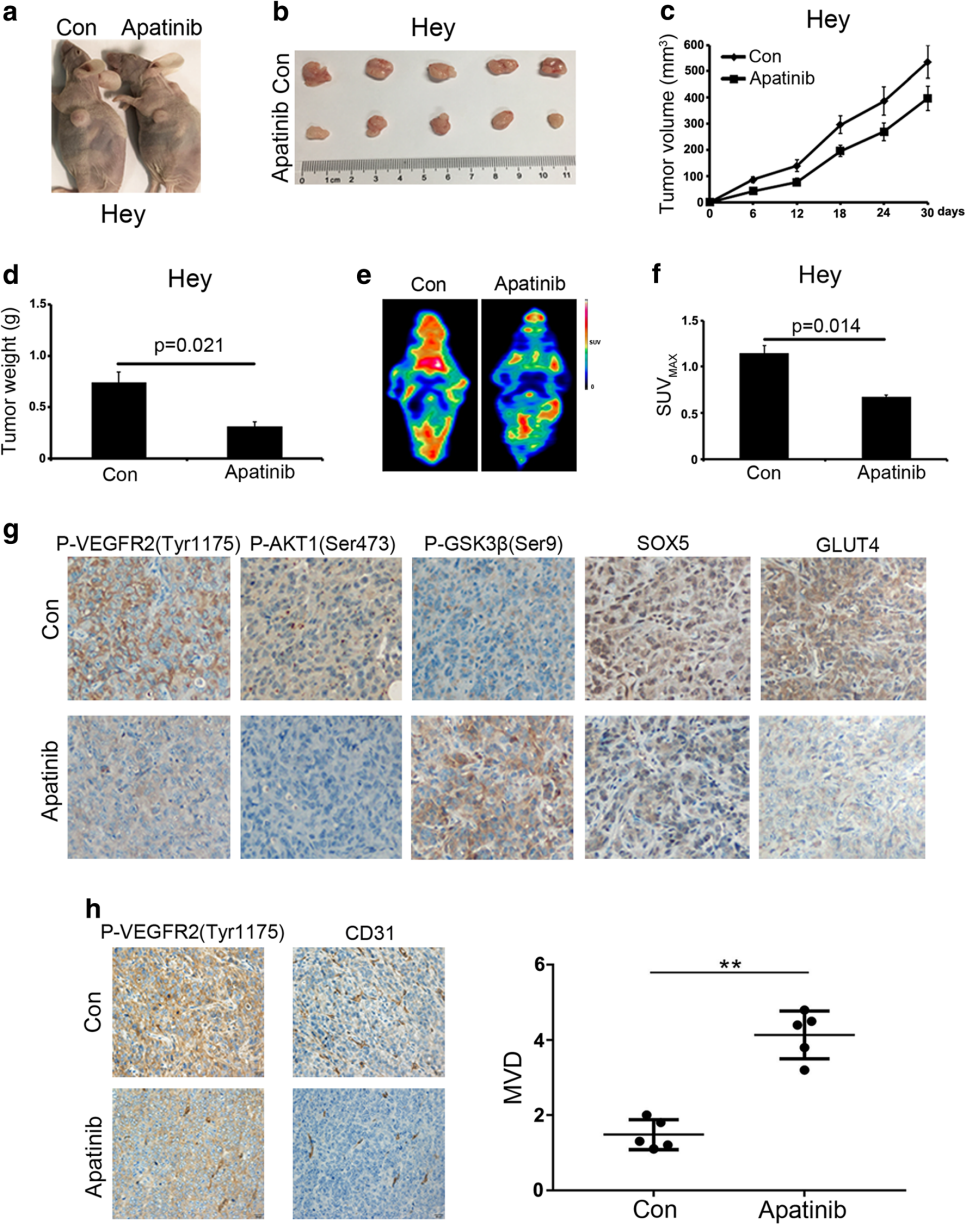
Apatinib inhibits in vivo proliferation and glycolysis of ovarian cancer cells. **a-b** Apatinib treatment slowed down in vivo Hey ovarian cancer cell tumor growth (*p* < 0.05). **c-d** Tumor volumes and tumor weights were significantly lower in the experimental group than in the control group (*p* < 0.05). **e-f** Apatinib significantly suppressed in vivo glucose uptake and resulted in a lower SUV (*p* < 0.05). **g** Immunohistochemical staining of xenografted tissues using antibodies directed against p-VEGFR2(Tyr1175), p-AKT1(Ser473), p-GSK3β(Ser9), SOX5 and GLUT4. **h** Blood vessel quantification in xenografted tumor tissues

To test the anti-tumor efficacy of apatinib in vivo, we subcutaneously inoculated Hey cells into nude mice for tumor formation. After the tumor volumes reached 100 mm^3^, the mice were intraperitoneally injected with apatinib (50 mg/kg). We found that the growth speed of the tumors in the apatinib treatment group was significantly slower than that of the control group (Fig. 5a-b). We also found that the tumor volumes and tumor weights in the drug-treated group were significantly reduced compared to those in the control group (*p* < 0.05, Fig. 5c-d). In addition, we found that, based on PET-CT results, apatinib treatment resulted in significantly suppressed in vivo glucose uptake in Hey tumor cells and in a lower SUVmax value (Fig. 5e-f). Subsequent IHC analyses using antibodies directed against p-VEGFR2(Tyr1175), p-AKT1(Ser473), p-GSK3β(Ser9), SOX5 and GLUT4 revealed that apatinib treatment decreased the expression of p-VEGFR2(Tyr1175), p-AKT1(Ser473), SOX5 and GLUT4, and increased the expression of p-GSK3β(Ser9) in the xenografted tumor tissues (Fig. 5g). We also assessed the expression of p-VEGFR2(Tyr1175) and CD31 (Platelet endothelial cell adhesion molecule-1, PECAM-1/CD31) by IHC in the xenografted tumor tissues and, by doing so, observed a decreased MVD (microvascular density) in the apatinib treatment group (Fig. 5h).

## Discussion

Here, we found that apatinib effectively suppressed cell proliferation in vitro and in vivo by regulating glycolysis through the VEGFR2/AKT1/GSK3β/SOX5 signaling pathway in ovarian cancer cells, providing a new perspective for the anti-tumor effect of apatinib. Aerobic glycolysis is an emerging hallmark of cancer [25, 26]. Glycolytic fueling generates energy and nutrients, thereby providing an appropriate microenvironment for cellular growth [27]. Therefore, targeting glycolysis is emerging as a novel therapeutic strategy [28]. Several drugs that specifically act on key molecules or enzymes in metabolic pathways, such as hexokinase (HK2) inhibitors, are currently under investigation or in clinical trials [29].

Apatinib is an oral anti-angiogenesis drug that selectively targets VEGFR2, with a binding affinity 10 times higher than that of vatalanib or sorafenib [30, 31], and in vitro experiments have revealed that apatinib may act as an even more selective VEGFR2 inhibitor than sunitinib, with an IC50 of 0.001 μM or 0.005 μM [14, 32]. Sunitinib is a multi-targeting agent directed against VEGFR-1, -2 and -3, platelet-derived growth factor receptor (PDGFR), c-Kit, FMS-like tyrosine kinase-3 and RET, and has been FDA approved for the treatment of renal cell cancer and gastrointestinal stromal tumors [32]. Recent clinical trials have shown that apatinib may benefit recurrent, platinum-resistant epithelial ovarian cancer patients with an acceptable safety profile [17]. In addition, it has been found that apatinib combined with oral etoposide shows a promising efficacy and manageable toxicity in platinum-resistant and platinum-refractory ovarian cancers [18]. Preclinical findings have indicated that apatinib may reverse multidrug resistance by inhibiting the transport function of the multidrug resistance protein1 (ABCB1), the multidrug resistance-associated protein 1(MRP1, ABCC1) and the breast cancer resistance protein (BCRP, ABCG2) [33–35]. Besides, there is ample evidence indicating that cellular metabolic disorders may be associated with drug resistance during cancer therapy [36, 37]. Apatinib has also been found to promote autophagy and apoptosis through the VEGFR2 signal transducer and activator of transcription 3 (STAT3)/B cell lymphoma 2 (BCL2) signaling pathway in osteosarcoma, and it has been found that a combined use of autophagy inhibitors can enhance the anti-tumor effects of apatinib [38]. Similarly, it has been shown that in colorectal cancer inhibition of autophagy can boost its efficacy, both in vitro and in vivo [39]. Therefore, we reasoned that apatinib may reverse multidrug resistance by inhibiting specific cellular functions, including glucose metabolism. Growing evidence indicates that apatinib may exert its anti-tumor effect and may gain efficacy when combined with other chemotherapeutics.

SOX5 is a member of the SOX family of transcription factors and plays a critical role in the progression of various cancer types. It has been reported that SOX5 may regulate epithelial-mesenchymal transition (EMT) via altering the expression of the twist-related protein 1 (Twist1) and, consequently, facilitating metastasis in prostate cancer [40]. Besides, it has been found that SOX5 overexpression may reverse apoptosis induced by miR-132 and miR-15a/16 in pituitary tumor cells and that SOX5 is upregulated in invasive pituitary tumor tissues [41]. These data suggest that SOX5 may act as a multifunctional oncogene, but its role in tumor initiation and progression needs to be further explored. We found that SOX5 regulates the promoter activity of GLUT4 to exert its effect on glycolysis.

In summary, our data indicate that apatinib may inhibit in vitro and in vivo glycolysis-induced cell growth and proliferation by suppressing the VEGFR2/AKT1/GSK3β/SOX5 signaling pathway. Our results provide new insights into the mechanism that underlies apatinib-regulated glycolysis, and offer opportunities for the design of novel approaches to improve the clinical outcome of recurrent, platinum-refractory ovarian cancer.

## Electronic supplementary material

Fig. S1 (PNG 1260 kb)

High Resolution Image (TIF 601 kb)

Table S1 (DOCX 17 kb)

Table S2 (DOCX 16 kb)
