# Supplementary figures and images for: Apatinib inhibits glycolysis by suppressing the VEGFR2/AKT1/SOX5/GLUT4 signaling pathway in ovarian cancer cells
Source: Cell Oncol (Dordr). 2019 Jul 20;42(5):679–90. doi: 10.1007/s13402-019-00455-x (PMC12994292; doi:10.1007/s13402-019-00455-x)

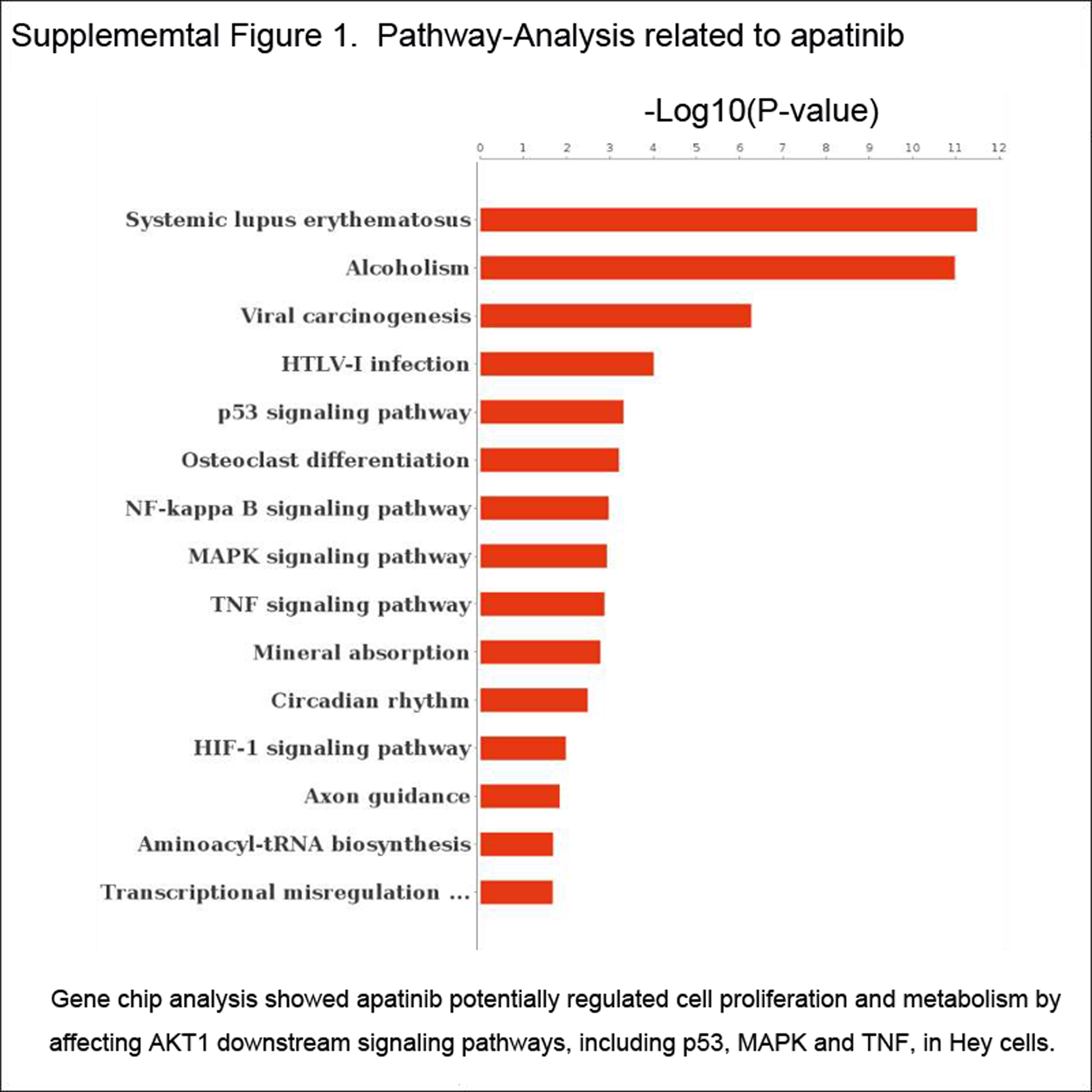

Supplement: Supplementary file 1 — (PNG 1260 kb) [file 13402_2019_455_Fig6_ESM.png]

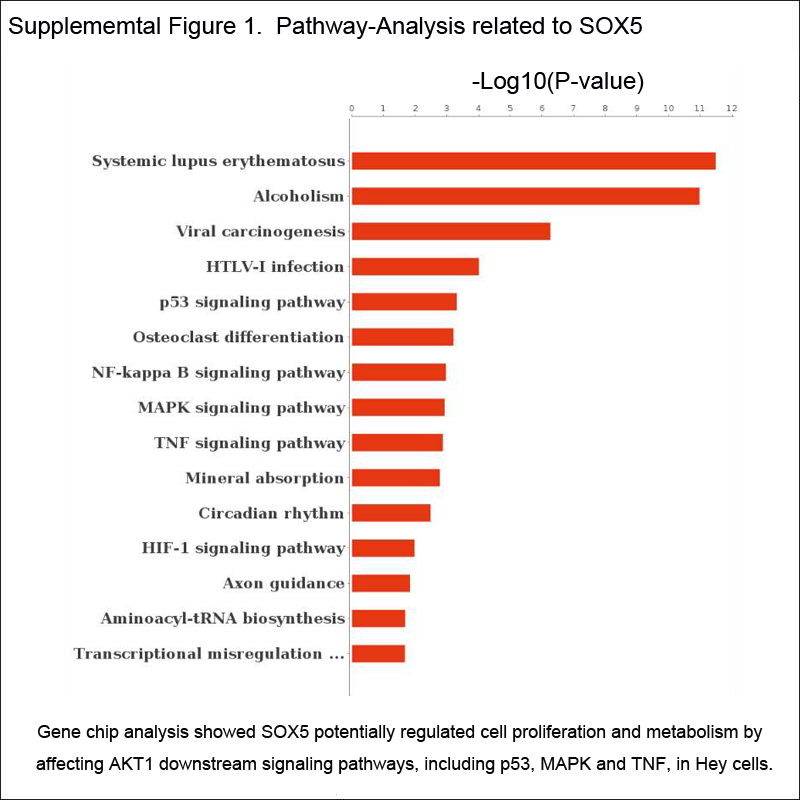

Supplement: Supplementary file 2 — High Resolution Image (TIF 601 kb) [file 13402_2019_455_MOESM1_ESM.tif]
