# Supplementary material for: Apatinib inhibits glycolysis by suppressing the VEGFR2/AKT1/SOX5/GLUT4 signaling pathway in ovarian cancer cells
Source: Cell Oncol (Dordr). 2019 Jul 20;42(5):679–90. doi: 10.1007/s13402-019-00455-x (PMC12994292; doi:10.1007/s13402-019-00455-x)
Supplement: Supplementary file 3 — (DOCX 17 kb) [file 13402_2019_455_MOESM2_ESM.docx]

**Supplemental Table 1. SOX5-target genes were enriched through GSEA analysis.**

|  | PROBE | GENE SYMBOL | GENE_TITLE | RANK IN GENE LIST | RANK METRIC SCORE | RUNNING ES | CORE ENRICHMENT |
| --- | --- | --- | --- | --- | --- | --- | --- |
| 1 | HHEX |  |  | 11183 | -0.732 | -0.4339 | Yes |
| 2 | HBP1 |  |  | 11196 | -0.735 | -0.4298 | Yes |
| 3 | NFIB |  |  | 11215 | -0.740 | -0.4261 | Yes |
| 4 | GLUT4 |  |  | 11224 | -0.742 | -0.4241 | Yes |
| 5 | UBR5 |  |  | 11234 | -0.746 | -0.4223 | Yes |
| 6 | FST |  |  | 11280 | -0.760 | -0.4206 | Yes |
| 7 | TP53 |  |  | 11331 | -0.776 | -0.4191 | Yes |
| 8 | CLIP2 |  |  | 11399 | -0.794 | -0.4188 | Yes |
| 9 | SEMA3A |  |  | 11470 | -0.811 | -0.4186 | Yes |
| 10 | RHOV |  |  | 11506 | -0.821 | -0.4157 | Yes |
| 11 | VAV3 |  |  | 11534 | -0.828 | -0.4120 | Yes |
| 12 | DENND2C |  |  | 11557 | -0.837 | -0.4080 | Yes |
| 13 | SEMA6C |  |  | 11671 | -0.879 | -0.4107 | Yes |
| 14 | ALDH1A2 |  |  | 11712 | -0.892 | -0.4076 | Yes |
| 15 | VSIG1 |  |  | 11727 | -0.895 | -0.4025 | Yes |
| 16 | EPHB6 |  |  | 11760 | -0.906 | -0.3988 | Yes |
| 17 | PELI2 |  |  | 11784 | -0.911 | -0.3943 | Yes |
| 18 | MLLT6 |  |  | 11804 | -0.918 | -0.3894 | Yes |
| 19 | CHM |  |  | 11806 | -0.918 | -0.3832 | Yes |
| 20 | DHRS3 |  |  | 11833 | -0.929 | -0.3788 | Yes |
| 21 | B3GALTL |  |  | 11843 | -0.934 | -0.3730 | Yes |

GSEA analysis showed that the expression levels of SOX5-target genes were down-regulated by the silencing of SOX5 in Hey cells.
