# Supplementary material for: Apatinib inhibits glycolysis by suppressing the VEGFR2/AKT1/SOX5/GLUT4 signaling pathway in ovarian cancer cells
Source: Cell Oncol (Dordr). 2019 Jul 20;42(5):679–90. doi: 10.1007/s13402-019-00455-x (PMC12994292; doi:10.1007/s13402-019-00455-x)
Supplement: Supplementary file 4 — (DOCX 16 kb) [file 13402_2019_455_MOESM3_ESM.docx]

**Supplemental Table 2. Sequences for primers.**

| **Name** | **Sequence (5’to 3’)** |
| --- | --- |
| GAPDH-F | GGCCTCCAAGGAGTAAGACC |
| GAPDH-R | CAAGGGGTCTACATGGCAAC |
| GLUT4-F | TGGAAGGAAAAGGGCCATGCTG |
| GLUT4-R | CAATGAGGAATCGTCCAAGGATG |
| HK2-F | GAGCCACCACTCACCCTACT |
| HK2-R | CCAGGCATTCGGCAATGTG |
| LDHA-F | ATGGCAACTCTAAAGGATCAGC |
| LDHA-R | CCAACCCCAACAACTGTAATCT |
| GLUT1-F | CTTTGTGGCCTTCTTTGAAGT |
| GLUT1-R | CCACACAGTTGCTCCACAT |
| G6P-F | GGTACACAGGCAAGACCATC |
| G6P-R | GTTTTGGCAATGTGAGTTCC |
| PFKL-F | CACAGGTGCCAACATCTTCCGCA |
| PFKL-R | TCATGTCGGTGCCGCAGAAGTCG |
| ALDOA-F | GTTATCAAATCCAAGGGCGGTGTT |
| ALDOA-R | AGTCAGCTCCGTCCTTCTTGTAC |
| PGK1-F | CAAGGTTAAAGCCGAGCCAGCCAA |
| PGK1-R | GCCTTCTGTGGCAGATTGACTCC |
| PGAM1-F | ATGATGTCCCACCACCTCCGAT |
| PGAM1-R | ATCCTTCAGACTCTCACAGGAG |
| ENO1-F | GCTCCGGGACAATGATAAGACTCG |
| ENO1-R | CTGTTCCATCCATCTCGATCATC |
| ENO2-F | TGAAGGCAGTGGACCACATCAACT |
| ENO2-R | AGAGACACACCCAGGATGGCATT |
| PKM2-F | CAAAGGACCTCAGCAGCCATGTC |
| PKM2-R | GGGAAGCTGGGCCAATGGTACAGA |
| SOX1-F | CAGTACAGCCCCATCTCCAAC |
| SOX1-R | GCGGGCAAGTACATGCTGA |
| SOX2-F | GCCGAGTGGAAACTTTTGTCG |
| SOX2-R | GGCAGCGTGTACTTATCCTTCT |
| SOX3-F | GACCTGTTCGAGAGAACTCATCA |
| SOX3-R | CGGGAAGGGTAGGCTTATCAA |
| SOX4-F | AGCGACAAGATCCCTTTCATTC |
| SOX4-R | CGTTGCCGGACTTCACCTT |
| SOX5-F | CAGCCAGAGTTAGCACAATAGG |
| SOX5-R | CTGTTGTTCCCGTCGGAGTT |
| SOX6-F | GGATGCAATGACCCAGGATTT |
| SOX6-R | TGAATGGTACTGACAAGTGTTGG |
